# Agrifood Waste Valorization: Development of Biochar from Peach Kernel or Grape Pits for Cr^6+^ Removal from Plating Wastewater

**DOI:** 10.3390/ma18174151

**Published:** 2025-09-04

**Authors:** Elena Raluca Cârjilă (Mihalache), Oanamari Daniela Orbuleț, Magdalena Bosomoiu, Cristina Modrogan, Eugenia Tanasă, Annette Madelene Dăncilă, Gabriel Gârleanu

**Affiliations:** 1 Department of Analytical Chemistry and Environmental Engineering, Faculty of Chemical Engineering and Biotechnologies, National University of Science and Technology Politehnica Bucharest, 7 Polizu Street, 011061 Bucharest, Romania; carjila.raluca@gmail.com (E.R.C.); oanamari.orbulet@upb.ro (O.D.O.); magdalena.bosomoiu@upb.ro (M.B.); madelene.dancila@upb.ro (A.M.D.); 2 Department of Physics, Faculty of Applied Sciences, National University of Science and Technology Politehnica Bucharest, 313 Splaiul Independenţei, 060042 Bucharest, Romania; eugenia.vasile@upb.ro; 3 Department of Quality Engineering and Industrial Technologies, Faculty of Industrial Engineering and Robotics, National University of Science and Technology Politehnica Bucharest, 313 Splaiul Independentei, 060042 Bucharest, Romania; gabriel.garleanu@upb.ro

**Keywords:** circular economy, eco-friendly sorbent, wastewater treatment, chromium plating, hexavalent chromium

## Abstract

In the context of circular economy, waste generated by fruit processing can be used to produce new materials with a wide range of uses. This study presents a method to synthesize biochar from peach kernel or grape pit waste. The adsorbents were tested in the removal of hexavalent chromium from synthetic wastewater with Cr^6+^ concentrations specific to plating processes. Characterization by BET, SEM, FTIR, and TG-DTG confirmed the formation of porous structures, and a well-functionalized surface. The effects of contact time, initial Cr^6+^ concentration, and adsorbent dose were investigated in static conditions. Both materials are efficient in hexavalent chromium removal, with sorption equilibrium achieved within 180 min. Kinetic studies indicated that the removal process follows a pseudo-second-order model. Equilibrium studies showed that optimal sorption occurred at pH = 6, with sorption capacities of 78.54 mg/g for biochar from peach kernels and 67.57 mg/g for biochar from grape pits. Hexavalent chromium followed a Sips adsorption isotherm for both biochars. Following the reusability study, it can be concluded that biochar from peach kernels maintains removal efficiency higher than 75% after four cycles.

## 1. Introduction

Water is one of the basic natural resources. Its geographical and seasonal distribution is uneven. Moreover, access to water resources is becoming problematic due to climate change. To remediate the water shortage, reclaimed water is used wherever possible, for example in industrial processes [1], agriculture [1], groundwater recharge [2], various urban uses and reintegration into water bodies used for drinking water production [3,4].

According to the WHO (World Health Organization), almost all hexavalent chromium sources are anthropogenic [5]. Depending on their provenance (e.g., plating for protection against corrosion [6], leather processing and manufacturing [7], textile dyeing [8], steel industry [9]), wastewater can be contaminated by hexavalent chromium. Chromium is an element that is found in the environment in its more stable forms: Cr^3+^ and Cr^6+^. While the trivalent form is a micronutrient for humans, hexavalent chromium is known to be toxic, affecting both humans and the environment [5,10].

The plating process consists of successive steps of surface chemical cleaning, washing and coating; these steps generate several types of wastewater with high concentrations of zinc, iron, grease or hexavalent chromium [11,12]. The present study focuses on the treatment of wastewater generated by chromium galvanizing baths.

In plating processes, the concentrations of Cr^6+^ range from 0.5 to 800 mg/L [6,11,13,14,15]; this is well above the limits for total chromium in water established by standards (e.g., 0.1 mg/L by EPA, 0.05 mg/L by the European Commission and WHO) [16,17,18]. The European Union plans to restrict the use of Cr^6+^ to prevent an estimated quantity of 17 tonnes of Cr^6+^ from being released in the environment [19].

The treatment of wastewater from plating consists of successive steps of pH adjustment, precipitation, sludge separation, sludge thickening and dewatering. All these steps use supplementary chemical reagents (e.g., FeCl_3_ ferric chloride, Na_2_S sodium sulfide, NaHSO_3_ sodium bisulfite, H_2_O_2_ hydrogen peroxide, etc.) [20]. The removal of pollutants by adsorption has several advantages compared with the previous methods: it is efficient and can achieve the fulfillment of concentration limits, does not require the addition of supplementary chemical reagents and has a low cost when biosorbents are used [21].

The common adsorbent, activated carbon, has been tested for the removal of hexavalent chromium from wastewater and has proven high adsorption capacity, but it has a high price [7]. To comply with the latest standards regarding waste management, researchers have started to investigate the manufacturing of valuable products from waste resulting from different activities. Biochar has been used in the formulation of sorbents tested for the removal of various pollutants from wastewater (e.g., pharmaceuticals [22], Cr^6+^ [23,24], heavy metals [25], lead [26], ammonia [27], Cd^2+^ and Ni^2+^ [28]). The Cr^6+^ removal efficiency for biochar materials was found to range between 80 and 99.79% [23,24]. In the case of Pb^2+^ removal, reusability studies indicated that the biochar removal efficiency decreases from 99.14% to 94.5% after four adsorption–desorption cycles, which makes biochar materials good candidates as sorbents [26]. Waste materials used in the production of sorbents offer multiple advantages: (i) waste minimization; (ii) obtaining a value-added product; (iii) replacement of high-cost adsorbents with cheap and efficient materials; (iv) the sorbent has low environmental risk. One of the biochar materials that has been tested for Cr^6+^ removal is obtained from coffee grounds [29]. A composite made from coffee ground biochar and alginate was found to be rich in oxygen-containing functional groups; it was evidenced that the material is not only a sorbent but also contributes to a toxicity decrease by hexavalent chromium’s reduction to trivalent chromium [24]. In regions where sugarcane bagasse by-product is produced in large quantities (e.g., India), research studies have proposed its transformation into biochar instead of dumping it on fields or burning [30]. In some cases, the biochar derived from agricultural waste was used for soil bioremediation [31]. Iron-enriched biochar was made from the sludge resulting from a wastewater treatment facility in the steel industry; the inclusion of iron in the formulation helps reduce hexavalent chromium to trivalent chromium [32]. However, because of its origin, the biochar is not suitable for producing high-purity water.

This study addresses a gap that exists in the circular economy in the agrifood branch by transforming waste into a valuable product that can be used for wastewater treatment. To the authors’ knowledge, waste from fruit processing has not been previously valorized in Cr^6+^ removal for plating wastewater treatment. Experimental and theoretical approaches have evidenced that biochars from peach kernels or grape pits are alternative sorbents for commercial materials and contribute to a reduction in agrifood waste generated by local businesses.

## 2. Materials and Methods

### 2.1. Chemicals and Reagents

K_2_Cr_2_O_7_ was used for the preparation of Cr^6+^ solution. Sulfuric acid was used in the preparation of the sorbent. Sodium hydroxide, hydrochloric acid, and sodium chloride were used for pH adjustment and pH_PZC_ determination. All chemical reagents were purchased from Merck/Sigma-Aldrich Chemical (Darmstadt, Germany). Ultrapure water used in the experiments was produced by the Milli-Q Integral system (Merck, Bucharest, Romania).

### 2.2. Preparation of Adsorbents

To prepare the peach kernel-derived biochar, peach kernels from the Peach Tree Black Boy variety were used. Grape seed-derived biochar was prepared from waste generated from the use of two grape varieties: large-seeded table grape seeds of the “Italia” variety and the “Afuz-Ali” variety. The raw material was purchased from a local market. The peach kernels or grape pits were washed thoroughly with tap water to remove impurities and dust and dried at room temperature for 48 h. Then they were ground to a particle size in the range of 1–3 mm using a BB51 jaw crusher (manufactured by Retsch, Haan, Germany). In a porcelain capsule, about 100 g of the shredded material was mixed with concentrated sulfuric acid at a mass ratio of 1:1, under slow stirring for 24 h. Then the solid material was recovered by filtration, it was washed with distilled water until the pH value was 6 and was placed in an oven at 140 °C for 8 h. The resulting powder was ground and stored in hermetically sealed containers.

### 2.3. Sample Characterization

FTIR spectra were recorded using a Bruker Vertex 70 spectrometer (Bruker, Billerica, MA, USA) equipped with a diamond ATR (attenuated total reflectance) device. The spectra were recorded in the range 600 to 4000 cm^−1^, as an average of 32 successive measurements, eliminating bands of noise and atmospheric carbon dioxide and water vapor.

TG and DTG analyses were carried out using STA 449 C Jupiter equipment from Netzsch (Gottinger, Germany), with a heating range from 25 to 800 °C at a constant heating rate of 10 °C/min and continuous air flow.

To understand the morphological structure of the biochars, scanning electron microscopy (SEM) was employed to visualize the sample surfaces. High-resolution images were recorded on a Hitachi S2600N (Chiyoda, Tokyo, Japan), which uses secondary electron imaging analysis (SEI) with a resolution of up to 4.0 nm (at 25 kV in high vacuum), and an energy-dispersive X-ray spectrometer (EDS) for qualitative and quantitative microanalysis.

The specific surface area measurements were performed by a nitrogen adsorption–desorption technique using a Micromeritics TriStar II Plus BET surface area analyzer (Malvern Panalytical, Malvern, Worcestershire, UK). For that, the samples were outgassed at 40 °C, 17 h before recording nitrogen adsorption–desorption isotherms. The specific surface area was determined in the relative pressure range P/P_0_ of 0.08–0.25 using the Brunauer–Emmett–Teller (BET) physical adsorption model.

### 2.4. Batch Experiments

Batch experiments were performed with synthetic solutions that contain hexavalent chromium in the concentration ranges specific to those found in plating wastewater. A volume of 50 mL of Cr^6+^ solution at the initial concentration *C*_0_ was mixed with various weights of biochar. The mixture was stirred at 200 rpm for a predetermined amount of time with a magnetic stirrer and placed in a thermostatic enclosure at 25 °C. Solutions of 0.1 M NaOH and 0.1 M HCl were used for pH adjustment. Kinetic experiments were made by taking a small volume of clear solution (0.1 mL) at regular intervals and determining the residual Cr^6+^ concentration. The method used for Cr^6+^ quantification consisted of supernatant analysis by flame atomic absorption spectroscopy (Analytik Jena ContrAA 300, Jena, Germany) at a wavelength of 357.9 nm.

The retention of Cr^6+^ from the aqueous solution was analyzed by calculating the sorption capacity q_t_, defined by

(1) $$q_{t}=\left(C_{0}-C_{t}\right)\times \frac{V}{m}$$

where C_0_ (mg·L^−1^) is the Cr^6+^ initial concentration; C_t_ (mg·L^−1^) is the Cr^6+^ residual concentration at the time t; q_t_ (mg·g^−1^) is the sorption capacity of biochar at time t; W (g) is the mass of the biochar; and V (L) is the volume of the Cr^6+^ solution.

The removal rate of Cr^6+^ ions was determined by calculating

(2) $$sorption\%=\frac{100(C_{0}-C_{t})}{C_{0}}$$

The determination of the point of zero charge was carried out according to the salt addition method [33]. A 0.1 M NaCl solution was used as a background electrolyte solution. Equal volumes (50 mL) of this solution were added to a series of flasks, and the pH was adjusted in the interval 1–11 using 0.1 M HCl and NaOH solutions. Afterwards, a constant weight of the sorbent (50 mg) was added to each flask. The final pH value was read after the samples were shaken at 200 rpm for 24 h in a temperature-controlled environment.

### 2.5. Stability Tests and Reusability

HCl 1M was used as a Cr^6+^ desorbent to explore the regeneration of the biochars over 4 adsorption–desorption cycles. For that, 50 mg of spent biochar was placed in a beaker with 50 mL of HCl 1 M; the mixture was slowly shaken at 50 rpm for 24 h. The biochar was separated by filtration and washed with deionized water until the pH of the filtrate was slightly acidic to neutral. The solid was then dried and reused.

To ensure experimental data reproducibility, every experiment was carried out three times, and the averages of the results were used in the further processing of the data.

## 3. Results

### 3.1. Characterization of Biochars

#### 3.1.1. Thermogravimetric Analysis of Adsorbent Materials

**Figure 1:**
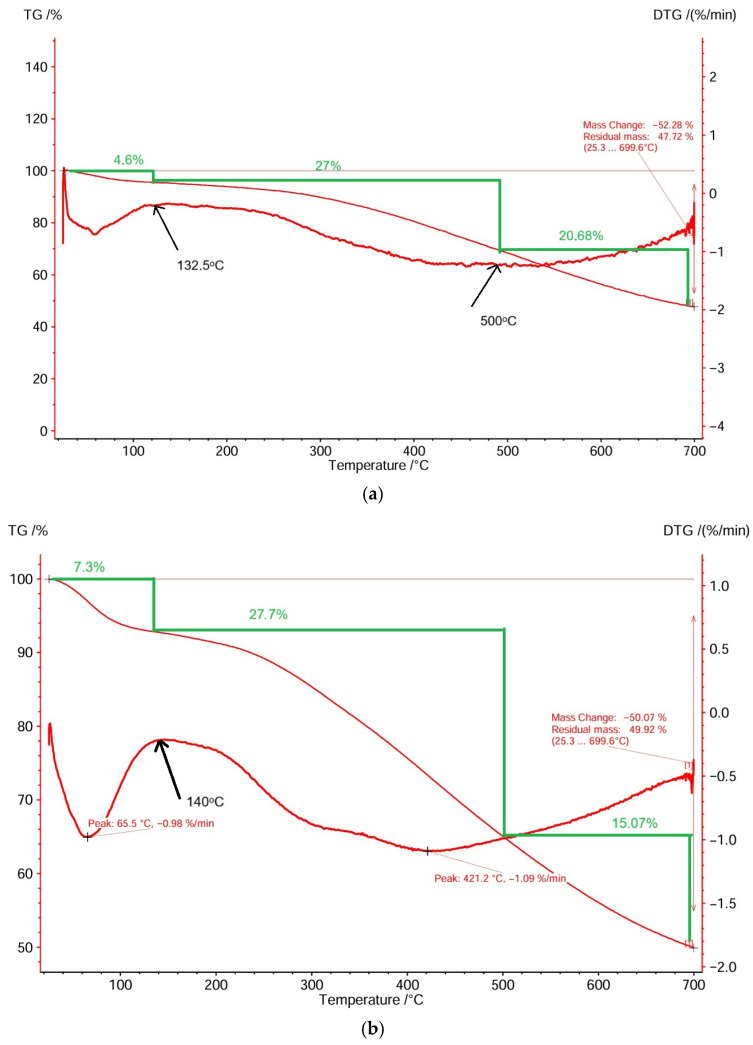
TG-DTG of biosorbents: (**a**) peach kernel biochar; (**b**) grape pit biochar.

The thermal behavior of the sorbents was studied using TG-DTG analyses. The total mass loss for peach kernel-derived biochar was 52.28% at 699.6 °C (Figure 1a), while grape pit-derived biochar had a total mass loss of 50.07% at 699.6 °C (Figure 1b). DTG graphs indicate that the decomposition of sorbents took place in three stages between 35 and 699.6 °C and between 32.5 and 699.6 °C, respectively. The first zone (35–132.5 °C and 32.5–140 °C, respectively) is characterized by an initial mass loss of 4.6% and 7.3%, respectively, corresponding to the evaporation of free water (endothermic peak in the DTG profiles). The main mass loss of 27% and 27.7%, respectively, was recorded during the second temperature interval (132.5–500 °C and 140–500 °C, respectively) and was attributed to the decomposition of less stable hemicellulose at temperatures below 350 °C [34] and cellulose at temperatures below 500 °C [35,36], and initiation of lignin decomposition [37]. The third temperature interval (500–699.6 °C) corresponds to a mass loss of 20.68% and 15.07%, respectively, due to the second-stage decomposition of the more stable polymer lignin [37,38].

Based on the thermogravimetric analysis of the samples, the biochars underwent evaporation of free water and only slight thermal decomposition of hemicellulose.

#### 3.1.2. Fourier Transform Infrared Spectroscopy

**Figure 2:**
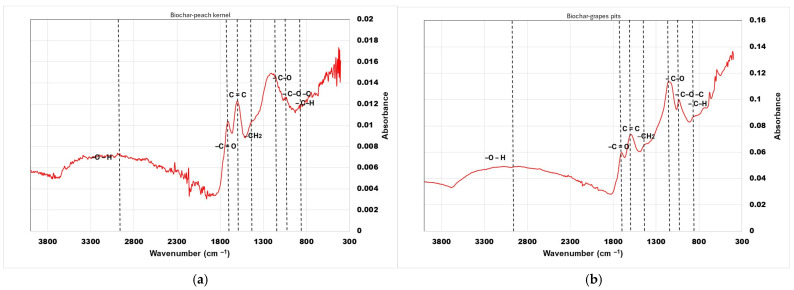
FT-IR results of biosorbents: (**a**) peach kernel biochar; (**b**) grape pit biochar.

The effect of thermal treatment on the surface functional groups of the sorbents was observed by FT-IR spectroscopy (Figure 2). The peaks at 2929 cm^−1^ (biochar from peach kernels) and 3011 cm^−1^ (biochar from grape pits) correspond to hydroxyl functional groups –OH (associated with alcohol, phenol, and water). Peaks at 1712 cm^−1^ and 1702 cm^−1^ correspond to carbonyl bond C=O stretching found in ketones, aldehydes, and esters. Peaks at 1598 cm^−1^ and 1594 cm^−1^ are attributed to C=C stretching ring vibrations in lignin. The bands at 1175 cm^−1^ and 1156 cm^−1^ indicate vibrations of C–O groups (ether or alcohol bonds), while vibrations at 1044 cm^−1^ and 1021 cm^−1^ indicate a C–O–C group (ether bond) or C–OH (alcohol group), respectively. The 858 cm^−1^ and 845 cm^−1^ peaks are generally associated with vibrations of C–H bonds in aromatic compounds.

#### 3.1.3. SEM and EDX Analysis

**Figure 3:**
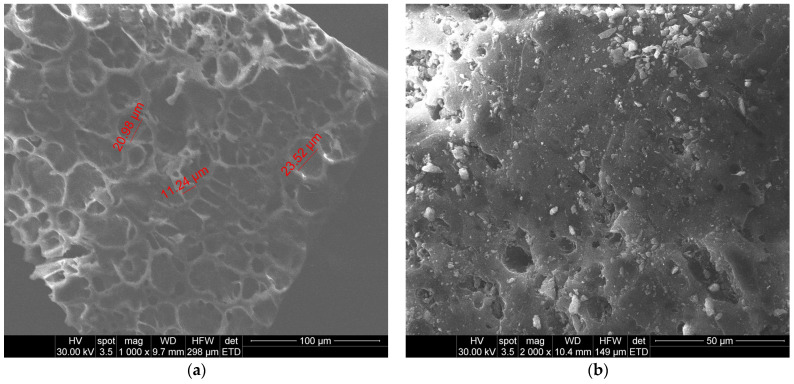
SEM results of biosorbents: (**a**) peach kernel biochar; (**b**) grape pit biochar.

The micrographs obtained by SEM analysis show that the biochar prepared from peach kernels has a spongy uniform surface and well-defined pores, while the second biochar presents smaller but less uniform pores (Figure 3).

The composition given by EDX analysis (Table 1) shows that the sorbents contain mainly carbon and oxygen and traces of other elements like sulfur, potassium, and calcium. The O to C ratio is 0.31 for biochar derived from peach kernels and 0.27 for the biochar derived from grape pits; this ratio is used to evaluate the degree of maturation of biochars [39], and indicates a higher content of aromatic compounds and enhanced carbonization for the second sorbent.

#### 3.1.4. Morphology Analysis and Specific Surface Area

Specific surface area is an important physical parameter; it was found that the composition (carbon percentage and the content of inorganic compounds) of biomass is a key factor that influences the specific surface area [40]. A comparison with data reported in the literature showed that the prepared materials have values of specific surface area comparable with those previously reported in the literature (Table 2).

#### 3.1.5. pH of Point of Zero Charge (pH_PZC_)

**Figure 4:**
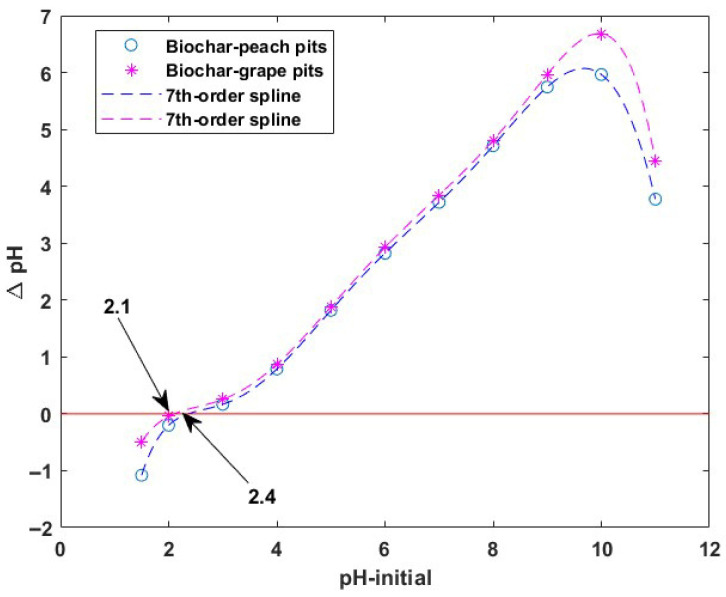
pH of zero charge (pH_PZC_) of the biochars.

The pH of the point of zero charge (pH_PZC_) of the tested sorbents was found to be 2.4 for biochar from peach kernels and 2.1 for biochar from grape pits (Figure 4). These values indicate that at a pH less than pH_PZC_, the biochar surface is positively charged, while at a pH higher than pH_PZC_, the surface is negatively charged. Thus, at pH values lower than 2.4 and 2.1, respectively, repulsive forces will appear between the positively charged surface of the sorbents and the hexavalent chromium ions. This is not favorable for the sorption process. These results are in agreement with data reported in the literature for biochar sorbents [42].

During this experiment it was noticed that at pH values higher than 11, chemical degradation of materials starts.

### 3.2. Batch Adsorption Tests

#### 3.2.1. Effect of m/V Ratio

**Figure 5:**
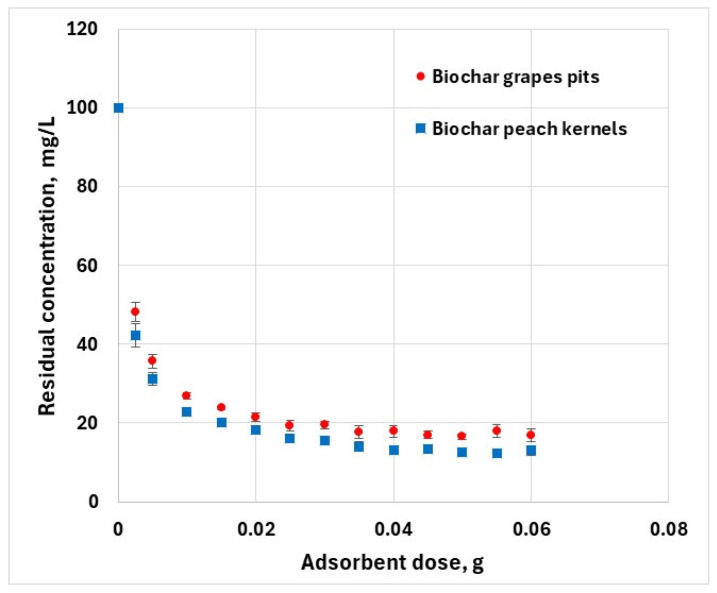
Effect of adsorbent dose on the removal of Cr^6+^ (*C*_0_ = 100 mg·L^−1^, *T* = 25 ± 2 °C; error bars show means ± standard error of the mean of triplicate experiments).

The ratio of adsorbent to adsorbate influences the pollutant removal efficiency. Various amounts of adsorbent were mixed with a fixed volume of adsorbate (50 mL) of 100 mg/L hexavalent chromium solution. The mixture was shaken for 10 h at a constant temperature. By increasing the adsorbent dose, the percentage of hexavalent chromium removed from aqueous solution increases from about 52% to 83% for grape pit biochar, and 57% to 87% for peach kernel biochar (Figure 5). This is because with increasing adsorbent quantity, the number of active sites available for adsorption increases. However, at higher adsorbent doses, a substantial portion of the adsorption sites remains unavailable because of the coalescence of solid particles.

#### 3.2.2. Effect of Contact Time

**Figure 6:**
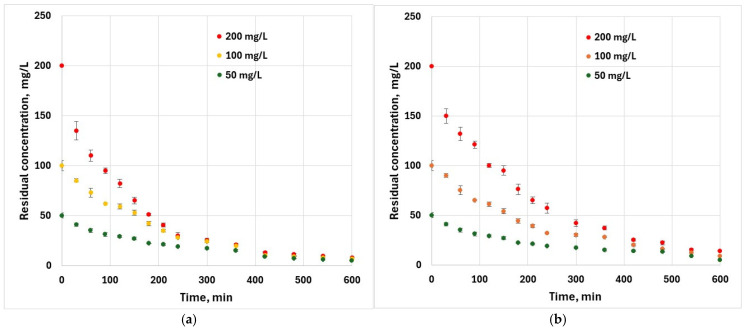
Effect of contact time on the removal of Cr^6+^ (*T* = 25 ± 2 °C, m = 0.05 g, V = 50 mL): (**a**) peach kernel biochar; (**b**) grape pit biochar.

The effect of contact time for an initial hexavalent chromium concentration of 200 mg·L^−1^ (Figure 6) indicates that initially, the sorption process is rapid and slows as equilibrium is approached. This is because at the beginning of the adsorption, a large number of adsorption sites were available while towards the equilibrium stage, this number reduces simultaneously with the appearance of repulsive forces between chromium ions in the external layer of the adsorbent particles.

#### 3.2.3. Effect of Temperature

**Figure 7:**
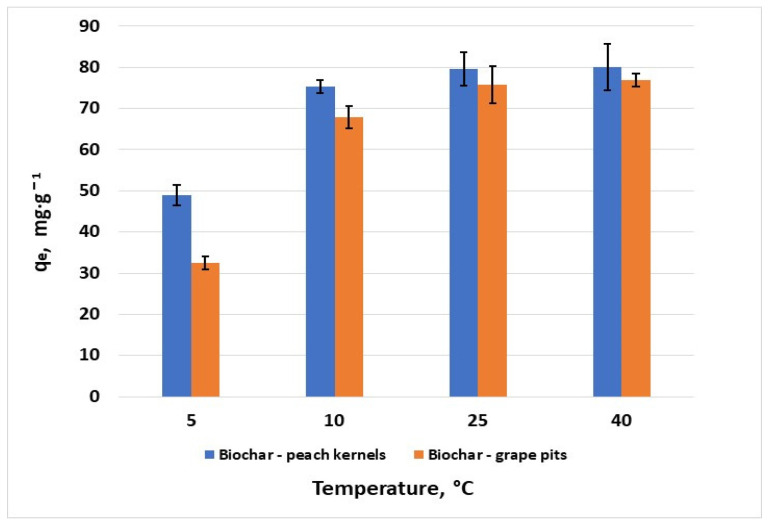
Effect of temperature on the removal of Cr^6+^ (*C*_0_ = 100 mg·L^−1^; error bars show means ± standard error of the mean of triplicate experiments).

The representation of temperature influence on hexavalent chromium removal (Figure 7) shows a slight increase in the adsorption capacity of both biochars in the range of 25 to 40 °C, with a maximum adsorption capacity of 76.9 mg·g^−1^ for grape pit biochar and 80 mg·g^−1^ for peach kernel biochar. This suggests an endothermic adsorption process, which is confirmed by other studies conducted on carbon-based adsorbents [30].

#### 3.2.4. Effect of pH

**Figure 8:**
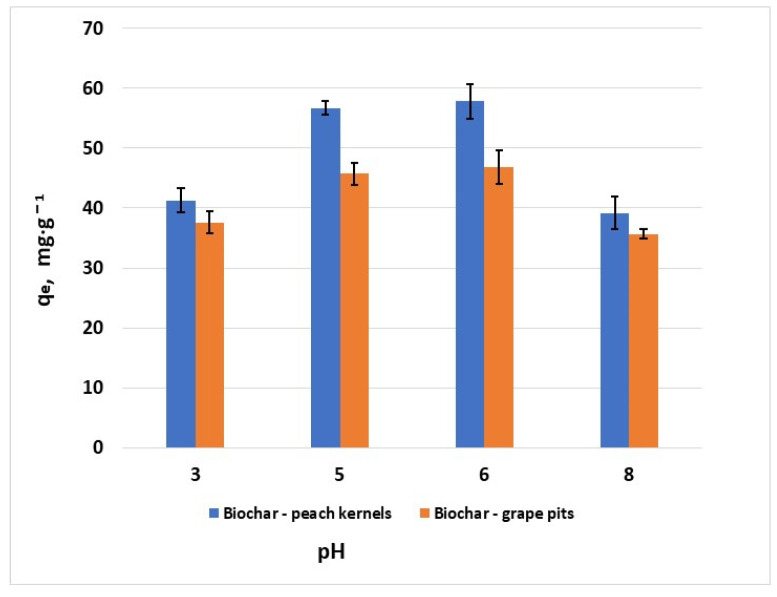
Effect of pH on the removal of Cr^6+^ (*C*_0_ = 100 mg·L^−1^, *T* = 25 ± 2 °C; error bars show means ± standard error of the mean of triplicate experiments).

Experiments at different pH values of the solutions indicated that increasing the pH leads to an increase in the adsorption capacity (Figure 8). This is attributed to the existence of electrostatic attractions between the hexavalent chromium ions and the negatively charged surface of the sorbents at pH values higher than pH_PZC_. The pH at which maximum hexavalent chromium adsorption capacity was observed was considered the optimum pH. Further experiments were performed at pH 6 for both biochar materials.

#### 3.2.5. Adsorption and Kinetic Models

##### Theoretical Backgrounds—Adsorption Models

The models usually used to describe the interaction between the sorbent and sorbate, at equilibrium and constant temperature, are Langmuir, Freundlich, Sips, Temkin, and Dubinin–Radushkevich. Reviewing the literature data, it was found that the best agreement between experimental and calculated data for biochar-type sorbents was obtained for chemical adsorption using the Langmuir isotherm and empirical Freundlich isotherm [43,44]. Additionally, Sips and Temkin models have been tested.

##### Langmuir Isotherm

The Langmuir model assumes equilibrium between the sorbate and the solid surface during adsorption, using hypotheses of monolayer sorption of the pollutant on the homogeneous surface of the biochar. The Langmuir isotherm’s non-linear form is described by Equation (3):

(3) $$q_{e}=q_{m}\frac{K_{L}C_{e}}{1+K_{L}C_{e}}$$

where q_e_ (mg·g^−1^) is the amount adsorbed at equilibrium concentration C_e_ (mg·L^−1^), q_m_ (mg·g^−1^) is the maximum amount of hexavalent chromium adsorbed per unit mass of biochar, and K_L_ (L·mg^−1^) is the Langmuir constant.

The ease of sorption can be analyzed by calculating the separation factor, R_L_:

(4) $$R_{L}=\frac{1}{1+K_{L}C_{0}}$$

The value of R_L_ indicates the type of isotherm: favorable (R_L_ < 1), unfavorable (R_L_ > 1), reversible (R_L_ = 0) and linear (R_L_ = 1).

##### Freundlich Isotherm

The Freundlich model is a non-linear, empirical model that is used when the Langmuir model fails to adequately describe the isothermal sorption process. This mechanism assumes that the sorbent surface is heterogeneous, which is closer to the characteristics of biochar materials, especially the biochar derived from grape pits (Figure 3b). It is represented by the following equation:

(5) $$q_{e}=K_{F}{C_{e}}^{1/n}$$

where K_F_ (g^−1^·mg^(1−1/n)^·L^1/n^) and n (dimensionless) are the Freundlich constants. n also indicates the nature of the adsorption process: when the value of 1/n is between 0 and 1, the adsorption is considered favorable, while a value of 1 simplifies the model to a simple linear one (q_e_ = K_F_·C_e_).

##### Sips Isotherm

(6) $$q_{e}=q_{m}\frac{K_{S}{C_{e}}^{1/ns}}{1+K_{S}{C_{e}}^{1/ns}}$$

where K_S_ (mg^−1/n^·L^1/n^) is the Sips constant that characterizes the affinity between the adsorbate and the adsorbent, and ns is a parameter that characterizes the surface heterogeneity of the sorbent. For ns = 1, the Sips model simplifies to the Langmuir model.

##### Temkin Isotherm

(7) $$q_{e}=\frac{RT}{b}\ln\left(K_{T}C_{e}\right)$$

where K_T_ (L·mg^−1^) is the adsorption equilibrium constant, T is the absolute temperature (K), R is the universal gas constant (8.314 J·mol^−1^·K^−1^), and b (mg·g^−1^·mol·J^−1^) is the Temkin constant related to the heat of adsorption.

##### Theoretical Backgrounds—Kinetic Models

The rate at which the hexavalent chromium ions are removed from the aqueous solution was investigated using the following models: pseudo-first-order, pseudo-second-order, Elovich, and intra-particle diffusion models, given by Equations (8)–(11).

##### Pseudo-First-Order Model

(8) $$q_{t}=q_{e}\left(1-e^{-k_{1}t}\right)$$

where k_1_ (min^−1^) stands for the rate constant of the pseudo-first-order model, and q_e_ (mg·g^−1^) and q_t_ (mg·g^−1^) are the removed amounts of Cr^6+^ at equilibrium and at time t (min) per unit mass of biochar (g).

##### Pseudo-Second-Order Model

(9) $$q_{t}=\frac{k_{2}{q_{e}}^{2}t}{1+k_{2}q_{e}t}$$

where k_2_ (g·mg^−1^·min^−1^) is the rate constant of the pseudo-second-order kinetic model.

##### Elovich Model

(10) $$q_{t}=\frac{1}{\beta }\ln\left(\alpha \beta t+1\right)$$

where α (mg·g^−1^·min^−1^) and β (g·mg^−1^) are kinetic parameters representing the initial adsorption rate and the desorption constant, respectively.

Because of computational simplicity, it is common to use the linearized form of the models described by Equations (3)–(10) [43]. However, linearization of these equations contributes to error propagation. Therefore, in this study, the estimation of adsorption parameters was made using the non-linear regression in Matlab R2024b. The goodness of the fitting was determined by calculating R^2^ (coefficient of determination), RMSE (root mean square error), and χ^2^ (chi-square).

#### 3.2.6. Thermodynamic Study

The thermodynamic study allows us to better understand the influence of temperature. The thermodynamic parameters were calculated by the following equations:

(11) $${\Delta G}_{T}^{0}={\Delta H}_{T}^{0}-{\Delta S}_{T}^{0}$$

(12) $${\Delta G}_{T}^{0}=-RT\ln K$$

(13) $$\ln K=-\frac{{\Delta H}_{T}^{0}}{RT}+\frac{{\Delta S}_{T}^{0}}{R}$$

where ${\Delta G}_{T}^{0}$ (J·mol^−1^) is the Gibbs free energy used to predict the spontaneity of the process, ${\Delta H}_{T}^{0}$ is the adsorption enthalpy (J·mol^−1^), and ${\Delta S}_{T}^{0}$ (J·mol^−1^) is used to determine the level of disorder of the adsorbate–adsorbent system. K is a non-dimensional equilibrium constant derived from the isotherm models (Equations (3)–(7)). Some studies report the direct use of equilibrium constants of one of the isotherms. However, this is not entirely correct because these have units. We have opted to correct the value of K with the equation proposed by Zhou et al. (2014) [45]; given the good fitting of experimental results by the Langmuir model, K_L_ was chosen as the equilibrium constant for the thermodynamic study:

(14) $$K=K_{L}M_{adsorbate}10^{3}\times 55.5$$

where M is the molecular weight. The isotherms were derived for three temperatures: 10, 25, and 40 °C.

## 4. Discussion

**Figure 9:**
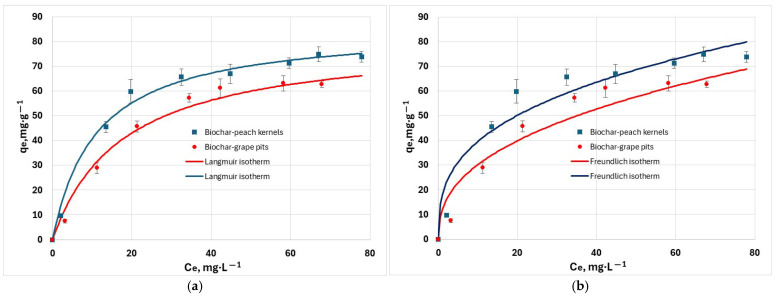

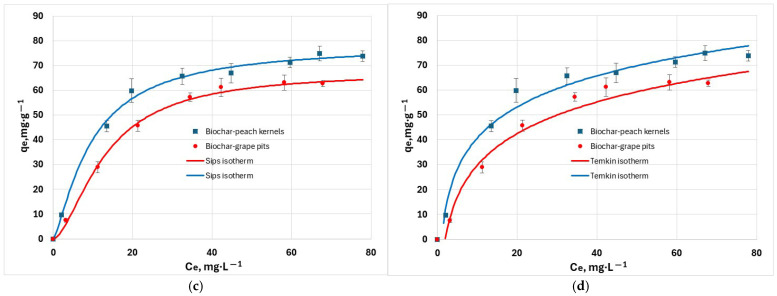
Adsorption isotherm of Cr^6+^ cations at 25 °C: (**a**) non-linearized Langmuir isotherm; (**b**) non-linearized Freundlich isotherm; (**c**) non-linearized Sips isotherm; (**d**) non-linearized Temkin isotherm.

The isotherm study gives information about the distribution of pollutant molecules between the liquid and solid phases. This study was performed by using initial hexavalent chromium concentrations in the range 100 to 250 mg/L, at 25 °C, pH = 6. The fitting results obtained for Langmuir, Freundlich, Sips, and Temkin isotherms (Figure 9) indicated strong sorption capacity of biochars for hexavalent chromium ions. Experimental data was fitted best to the Sips isotherm than to the other models, as indicated by the RMSE and χ ^2^ values in Table 3. The R^2^ criterion is accurate only for linear models. Hence, it was regarded only for orientation because the models were solved in their non-linear form. The R_L_ parameter is 0.78 for biochar from grape waste, and 0.68 for biochar from peach waste, indicating that the adsorption process is favorable.

Sips adsorption capacities were compared with data from the literature for low-cost adsorbents; as shown in Table 4, the synthesised biochars have comparable adsorption capacities or better.

**Figure 10:**
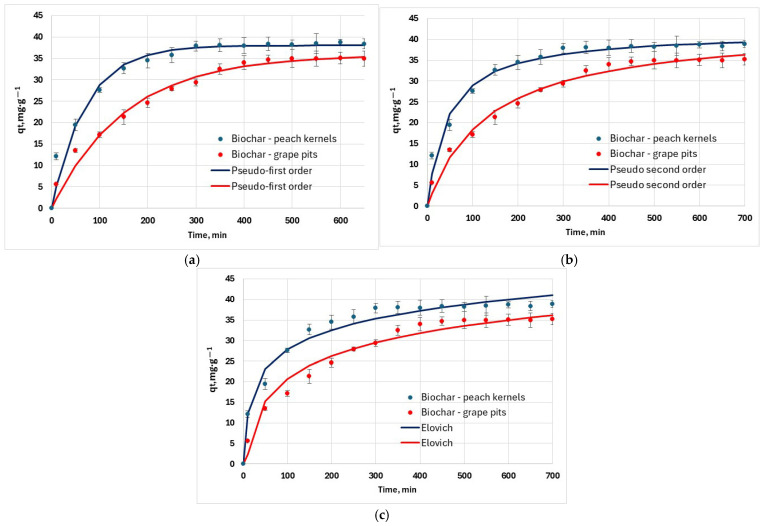
Adsorption kinetic models used to fit experimental data (25 °C): (**a**) pseudo-first-order kinetic model; (**b**) pseudo-second-order kinetic model; (**c**) Elovich model.

Kinetic experimental data was obtained by the variation of interaction time (Figure 10a). Data was fitted against the pseudo-first-order, pseudo-second-order, and Elovich kinetic models (Figure 10). Determination coefficients for the kinetic models have values above 0.9, indicating a good correlation between experimental and calculated data (Table 5). However, R^2^ alone is not enough to discriminate between the kinetic models. In the case of both adsorbents, considering the values of RMSE and χ^2^, the kinetic experimental data match the pseudo-second-order model well (Figure 10b).

The thermodynamic parameters are presented in Table 6. The negative values of the Gibbs free energy demonstrate the spontaneous nature of the adsorption on the two sorbents. It decreases with increasing temperature; this indicates the feasibility of adsorption at increasing temperature. The positive values of the enthalpy indicate that the adsorption process is endothermic, and the interactions between the adsorbate and the adsorbent are predominantly through weak van der Waals forces (physical adsorption). The positive values of the entropy indicate increased disorder at the adsorbent–solution interface containing hexavalent chromium.

## 5. Regeneration and Reusability

**Figure 11:**
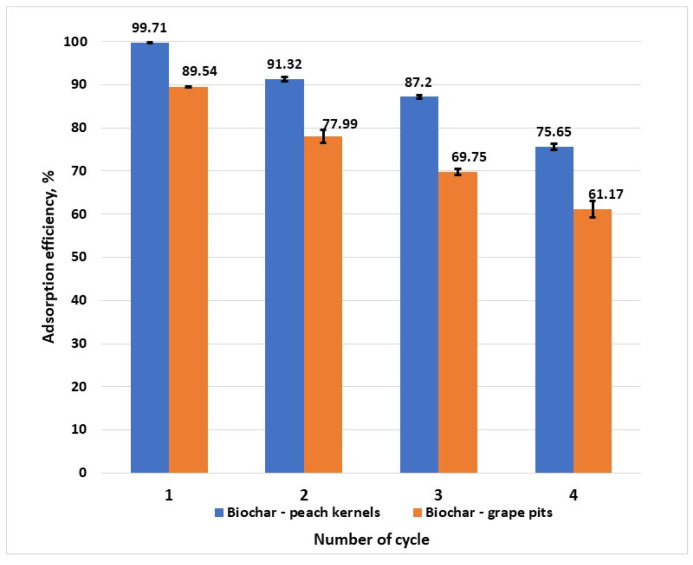
Adsorbent adsorption–desorption cycles and regeneration experiment (C_0_ =100 mg/L, T = 25 ± 2 °C).

From the practical point of view, the regeneration and reusability of an adsorbent are decisive factors that determine the long-term applicability of the material. Adsorption–desorption experiments showed that biochar from peach kernels maintains a removal efficiency over 75% after four cycles (Figure 11), while biochar from grape pits showed a faster aging of the structure and a removal efficiency of 77.99% after the second cycle. The decrease in removal efficiency can be attributed to the gradual occupation of active sites with hexavalent chromium ions that are strongly bound, as well as to the structural changes. Nevertheless, the biochar from peach kernels has the ability to maintain high Cr^6+^ removal efficiency over multiple cycles.

Regarding the possible secondary contamination from the sorbent, carbon-based adsorbents are used worldwide in water and wastewater treatment applications. They are well known for their stability and for not releasing dangerous compounds in the normal operating conditions (neutral pH, slightly acidic or basic, ambient temperature, etc).

## 6. Conclusions

Biochar materials were synthesized from agricultural waste (peach kernels or grape pits). The obtained materials were characterized by BET, SEM, FTIR, and TG-DTG. The results indicated a uniform porous surface for biochar from peach kernels while the surface of biochar from grape pits was found to be uneven. FT-IR data indicated a good functionalization of the adsorbents’ surfaces.

The adsorption equilibrium of hexavalent chromium was investigated by Langmuir, Freundlich, Sips, and Temkin adsorption isotherm models. The Sips model was best for representing the equilibrium adsorption of hexavalent chromium on both biochars. The maximum adsorption capacities were found to be 78.54 mg/g for biochar from peach kernels and 67.57 mg/g for biochar from grape pits, according to the Sips model. According to data reported in the literature, these values are in the upper range for low-cost sorbent materials used for hexavalent chromium removal.

The pseudo-second-order kinetic model was found to be the best option for interpreting the kinetics of hexavalent chromium removal by sorption on both biochars.

The reusability study indicates that the biochars can be reused for at least four adsorption–desorption cycles.

## Figures and Tables

**Table 1:** EDX analysis results of biochars.

Elements	Biochar from Peach Kernels Atomic Percentage (%)	Biochar from Grape Pits Atomic Percentage (%)
**C**	76.05	77.23
O	23.83	21.09
S	0.11	1.36
K	-	0.09
Ca	-	0.23

**Table 2:** Specific surface area and porosity of prepared samples and values from the literature.

Material	S_BET_ (m^2^·g^−1^)	V_t_ (cm^3^·g^−1^)	Reference
Coffee ground biochar 400	9.29	0.009	[24]
Coffee ground biochar 700	4.95	0.007	[24]
Rice straw biochar	6.96	0.011	[26]
Walnut shell biochar	5.75	-	[27]
Pine biochar	1.24	0.097	[41]
Peach kernel biochar	0.344	0.003	[28]
Peach kernel biochar	4.4	0.007	Present work
Grape pit biochar	6.0	0.004	Present work

**Table 3:** Parameters of isothermal adsorption models for hexavalent chromium removal at 25 °C.

Model	Unit	Biochar—Peach Kernels	Biochar—Grape Pits
Langmuir			
q_m_	mg∙g^−1^	85.8	81.2
K_L_	L∙mg^−1^	9.04 × 10^−2^	5.64 × 10^−2^
R_L_		0.68	0.78
R^2^		0.992	0.984
RMSE		2.43	3.02
χ^2^		1.81	3.03
Freundlich			
K_F_	g^−1^·mg^(1−1/n)^·L^1/n^	17.97	11.95
1/n		0.342	0.402
R^2^		0.942	0.929
RMSE		6.38	6.30
χ^2^		11.10	11.11
Sips			
q_m_	mg∙g^−1^	78.54	67.57
K_S_	mg^−1/n^·L^1/n^	5.02 × 10^−2^	1.42 × 10^−2^
n_S_		0.758	0.603
R^2^		0.996	0.997
RMSE		1.73	1.18
*χ* ^2^		0.48	0.56
Temkin			
b	mg·g^−1^·mol·J^−1^	137.37	134.99
K_T_	L·mg^−1^	9.56 × 10^−1^	5.06 × 10^−1^
R^2^		0.973	0.964
RMSE		3.33	3.62
χ^2^		2.03	2.06

**Table 4:** Adsorption capacity of several adsorbents for Cr^6+^ removal.

Sorbent	Adsorption Capacity (mg·g^−1^)	Reference
Palm shell		[46]
PEI/activated carbon	20.5	
Activated carbon (AC)	12.6	
AC from bael fruit shell	17.3	[47]
Waste AC	10.9	[48]
Magnetic biochar (modified by Fe_3_O_4_ @ SiO_2_—NH_2_ particles)	27.2	[49]
Biochar—iron composite	81.5	[23]
LDH—Al	17.0	[50]
Biochar from peach kernels	78.54	Present work
Biochar from grape pits	67.57	Present work

**Table 5:** Kinetic parameters for hexavalent chromium removal by biochars at 25 °C.

Model	Unit	Biochar—Peach Kernels	Biochar—Grape Pits
q_e_—experimental	mg∙g^−1^	38.9	35.2
Pseudo-first-order			
q_e_	mg∙g^−1^	38.03	35.92
k_1_	min^−1^	1.42 × 10^−1^	6.49 × 10^−3^
R^2^		0.971	0.983
RMSE		1.94	1.45
χ^2^		10.09	6.53
Pseudo-second-order			
q_e_	mg∙g^−1^	41.80	43.39
k_2_	g·mg^−1^·min^−1^	5.43 × 10^−4^	1.70 × 10^−4^
R^2^		0.964	0.980
RMSE		1.48	1.28
χ^2^		2.98	3.09
Elovich			
α	mg·g^−1^min^–1^	4.106	1.073
β	g∙mg^−1^)	1.47 × 10^−1^	1.25 × 10^−1^
R^2^		0.948	0.958
RMSE		1.77	1.85
*χ* ^2^		1.52	6.07

**Table 6:** Thermodynamic parameters for hexavalent chromium adsorption at *C_0_*=100 mg/L.

Material	∆*G*° (kJ/mol)			∆*H*° (kJ/mol)	∆*S*° (J/K/mol)
	283 K	298 K	313 K		
Biochar—peach kernels	−29.9	−30.9	−32.1	27.2	73.8
Biochar—grape pits	−29.1	−29.7	−30.8	19.9	63.1

## Data Availability

The original contributions presented in this study are included in the article. Further inquiries can be directed to the corresponding author.

## References

[1] Palumbo M., Carbone V., Ricci I., Pace B., Cefola M., Minasi P., Garofalo S.P., Camposeo S., Tallou A., Vivaldi G.A. (2024). Qualitative and biochemical characteristics of pomegranate fruit grown using reclaimed water and low input fertigation treatments at harvest and during storage. Heliyon.

[2] Sifi S., Aydi A., Zaghdoudi S., Gasmi M., Abdo H.G. (2025). Geospatial technique and multi-criteria evaluation to select suitable sites for groundwater recharge with reclaimed water in arid and semi-arid regions. Water Cycle.

[3] Dogan S., Azarm S.A.S. (2024). Cost analysis and site selection for reclaimed water injection to enhance coastal aquifer sustainability. J. Water Process Eng..

[4] Recycled Water for Drinking: An Overview 2024. https://www.cdc.gov/drinking-water/about/recycled-water-for-drinking-an-overview.html.

[5] WHO Environmental Health Criteria 61: Chromium. https://iris.who.int/bitstream/handle/10665/40419/9241542616-eng.pdf?sequence=1&isAllowed=y.

[6] Bratovcic A., Buksek H., Helix-Nielsen C., Petrinic I. (2022). Concentrating hexavalent chromium electroplating wastewater for recovery and reuse by forward osmosis using underground brine as draw solution. Chem. Eng. J..

[7] Jimenez-Paz J., Lozada-Castro J.J., Lester E., Wiliams O., Stevens L., Barraza-Burgos J. (2023). Solutions to hazardous wastes issues in the leather industry: Adsorption of Chromium iii and vi from leather industry wastewaters using activated carbons produced from leather industry solid wastes. J. Environ. Chem. Eng..

[8] Singha K., Pandit P., Maity S., Sharma S.R. (2021). Harmful environmental effects for textile chemical dyeing practice. Green Chemistry for Sustainable Textiles.

[9] Rawat A., Srivastava A., Bhatnagar A., Gupta A.K. (2023). Technological advancements for the treatment of steel industry wastewater: Effluent management and sustainable treatment strategies. J. Clean. Prod..

[10] Vaiopoulou E., Gikas P. (2020). Regulations for chromium emissions to the aquatic environment in Europe and elsewhere. Chemosphere.

[11] Hackbarth F.V., Maass D., de Souza A.A.U., Vilar V.J.P., de Souza S.M.A.G.U. (2016). Removal of hexavalent chromium from electroplating wastewaters using marine macroalga *Pelvetia canaliculata* as natural electron donor. Chem. Eng. J..

[12] Santis A., Arbeláez O., Cardenas L.A., Castellanos J., Velasquez P. (2024). Optimizing Cr(VI) Reduction in Plastic Chromium Plating Wastewater: Particle Size, Irradiation, Titanium Dose. Emerg. Sci. J..

[13] Erkabaev F., Muhammadieva D., Rabbimkulova S. (2024). Composition and properties of industrial wastewater and its electrochemical treatment. E3S Web Conf..

[14] Kobya M., Erdem N., Demirbas E. (2015). Treatment of Cr, Ni and Zn from galvanic rinsing wastewater by electrocoagulation process using iron electrodes. Desalination Water Treat..

[15] Rodríguez R., Espada J.J., Gallardo M., Molina R., Lopez-Munoz M.J. (2018). Life cycle assessment and techno-economic evaluation of alternatives for the treatment of wastewater in a chrome-plating industry. J. Clean. Prod..

[16] EPA Chromium in Drinking Water. https://www.epa.gov/sdwa/chromium-drinking-water#what-are-regs.

[17] Council Directive 98/83/EC of 3 November 1998 on the Quality of Water Intended for Human Consumption. https://eur-lex.europa.eu/eli/dir/1998/83/oj/eng.

[18] WHO Chromium in Drinking-Water 2003. https://cdn.who.int/media/docs/default-source/wash-documents/wash-chemicals/chromium.pdf?sfvrsn=37abd598_6.

[19] ECHA Proposes Restrictions on Chromium (VI) Substances to Protect Health. https://echa.europa.eu/-/echa-proposes-restrictions-on-chromium-vi-substances-to-protect-health.

[20] Tumolo M., Ancona V., De Paola D., Losacco D., Campanale C., Massarelli C., Uricchio V.F. (2020). Chromium Pollution in European Water, Sources, Health Risk, and Remediation Strategies: An Overview. Int. J. Environ. Res. Public Health.

[21] Kerur S.S., Bandekar S., Hanagadakar M.S., Nandi S.S., Ratnamala G.M., Hegde P.G. (2021). Removal of hexavalent Chromium-Industry treated water and Wastewater: A review. Mater. Today Proc..

[22] Ndankou C.S.D., Ștefan D.S., Nsami N.J., Daouda K., Bosomoiu M. (2024). Evaluation of Phenobarbital Adsorption Efficiency on Biosorbents or Activated Carbon Obtained from Adansonia Digitata Shells. Materials.

[23] Sun Y., Lyu H., Gai L., Sun P., Shen B., Tang J. (2023). Biochar-anchored low-cost natural iron-based composites for durable hexavalent chromium removal. Chem. Eng. J..

[24] Tian Y., Sun X., Chen N., Cui X., Yu H., Feng Y., Xing D., He W. (2024). Efficient removal of hexavalent chromium from wastewater using a novel sodium alginate-biochar composite adsorbent. J. Water Process Eng..

[25] Burlacu I.F., Deák G., Favier L., Serre I.P., Balloy D. (2021). Advanced Catalytic Materials Obtained From Waste For Wastewater Treatment Applications. Univ. Polytech. Buchar. Sci. Bull. Ser. B.

[26] Tan G., Wu Y., Liu Y., Xiao D. (2025). Removal of Pb(II) ions from aqueous solution by manganese oxide coated rice straw biochar—A low-cost and highly effective sorbent. J. Environ. Chem. Eng..

[27] Yu Y., An Q., Li Z., Zhao B. (2024). Synergistic removal of ammonia nitrogen and hexavalent chromium by the hybrid-system of Acinetobacter baumannii AL-6 and original walnut shell biochar in solution: Mechanism and application. J. Environ. Chem. Eng..

[28] Bian Y., Zhang F., Liu Q., Mo X., Xu T., Yi W., Xu Y., Bai S., Liu L. (2024). Simultaneous removal capacity and selectivity of Cd(II) and Ni(II) by KMnO4 modified coconut shell and peach kernel biochars. J. Water Process Eng..

[29] Campbell R., Xiao B., Mangwandi C. (2024). Production of activated carbon from spent coffee grounds (SCG) for removal of hexavalent chromium from synthetic wastewater solutions. J. Environ. Manag..

[30] Mondal G., Sahoo P., Banerjee S., Nandi R., Ghosh C., Mandal J., Bhattacharyya P. (2025). Utilizing nano zero-valent iron impregnated biochar for removal of hexavalent chromium from water: An assessment through Box-Behnken optimization, kinetics, and isotherm studies. Groundw. Sustain. Dev..

[31] Wang X., Zhao R., Wu H., Jia X., Liu Y., Zhou G., Chen S., Zhao F., Li L., Hu S. (2024). Enhanced bioremediation of hexavalent chromium via *Stenotrophomonas acidaminiphila* 4–1 assisted with agricultural wastes-derived biochar. Biochem. Eng. J..

[32] Zeng Y., Zhou l., Wang X., Zhang G., Bao X., Yan Z., Ma W. (2025). One-step synthesis of iron-rich biochar for efficient hexavalent chromium removal: Adsorption-reduction performance, mechanism and column experiments. J. Environ. Chem. Eng..

[33] Mehrabi N., Soleimani M., Yeganeh M.M., Sharififard H. (2015). Parameter optimization for nitrate removal from water using activated carbon and composite of activated carbon and Fe_2_O_3_ nanoparticles. RSC Adv..

[34] Waters C.L., Janupala R.R., Mallinson R.G., Lobban L.L. (2017). Staged thermal fractionation for segregation of lignin and cellulose pyrolysis products: An experimental study of residence time and temperature effects. J. Anal. Appl. Pyrolysis.

[35] Bernardino C.A.R., Mahler C.F., Veloso M.C.C., Romeiro G.A. (2017). Preparation of Biochar from Sugarcane By-product Filter Mud by Slow Pyrolysis and Its Use Like Adsorbent. Waste Biomass Valorization.

[36] Wang J., Minami E., Asmadi M., Kawamoto H. (2021). Effect of delignification on thermal degradation reactivities of hemicellulose and cellulose in wood cell walls. J. Wood Sci..

[37] López-Beceiro J., Díaz-Díaz A.M., Álvarez-García A., Tarrío-Saavedra J., Naya S., Artiaga R. (2021). The Complexity of Lignin Thermal Degradation in the Isothermal Context. Processes.

[38] Jagnade P., Panwar N.L., Agarwal C. (2023). Experimental Investigation of Kinetic Parameters of Bamboo and Bamboo Biochar Using Thermogravimetric Analysis Under Non-Isothermal Conditions. Bioenergy Res..

[39] Ma X., Zhou B., Budai A., Jeng A., Hao X., Wei D., Zhang Y., Rasse D. (2016). Study of Biochar Properties by Scanning Electron Microscope—Energy Dispersive X-Ray Spectroscopy (SEM-EDX). Commun. Soil Sci. Plant Anal..

[40] Leng L., Xiong Q., Yang L., Li H., Zhou Y., Zhang W., Jiang S., Li H., Huang H. (2021). An overview on engineering the surface area and porosity of biochar. Sci. Total Environ..

[41] Dudło A., Michalska J., Turek-Szytow J., Kobyłecki R., Zarzycki R., Wichlinski M., Surmacz-Gorska J. (2024). Humic substances sorption from wastewater on the biochar produced from the waste materials. J. Environ. Manag..

[42] Kasera N., Augoustides V., Kolar P., Hall S.G., Vicente B. (2022). Effect of Surface Modification by Oxygen-Enriched Chemicals on the Surface Properties of Pine Bark Biochars. Processes.

[43] Wang J., Guo X. (2020). Adsorption isotherm models: Classification, physical meaning, application and solving method. Chemosphere.

[44] Saleh T.A. (2022). Isotherm models of adsorption processes on adsorbents and nanoadsorbents. Interface Sci. Technol..

[45] Zhou X., Zhou X. (2014). The unit problem in the thermodynamic calculation of adsorption using the Langmuir equation. Chem. Eng. Commun..

[46] Owlad M., Aroua M.K., Daud W.A.W. (2010). Hexavalent chromium adsorption on impregnated palm shell activated carbon with polyethyleneimine. Bioresour. Technol..

[47] Anandkumar J., Mandal B. (2009). Removal of Cr(VI) from aqueous solution using Bael fruit (*Aegle marmelos* correa) shell as an adsorbent. J. Hazard. Mater..

[48] Ghosh P.K. (2009). Hexavalent chromium [Cr(VI)] removal by acid modified waste activated carbons. J. Hazard. Mater..

[49] Shi S., Yang J., Liang S., Li M., Gan Q., Xiao K., Hu J. (2018). Enhanced Cr(VI) removal from acidic solutions using biochar modified by Fe_3_O_4_@SiO_2_-NH_2_ particles. Sci. Total Environ..

[50] Li Z., Fang X., Yuan W., Zhang X., Yu J., Chen J., Qiu X. (2023). Preparing of layered double hydroxide-alginate microspheres for Cr(VI)-contaminated soil remediation. Colloids Surf. A Physicochem. Eng. Asp..

